# Who Can the Lady Be?

**Published:** 1919-07-05

**Authors:** 


### THE EDITOR'S LETTER-BOX.

**[Correspondence on all subjects is invited, but we cannot in any way be responsible for the opinions expressed by our correspondents, who must give their name and address as a guarantee of good faith, but not necessarily for publication. Correspondents are reminded that brevity of style and conciseness of statement greatly facilitate early insertion.]**

### Who Can the Lady Be?

*To the Editor of* THE HOSPITAL.

SIR,—A public man recently received the following request by letter:

"Will you forgive my venturing to ask you to consider whether the incident to which you referred in your opening remarks might be omitted from your speech? The lady in question is at this moment very much in the same position as Germany—who, in impotent despair of not being able to obtain what she wants, is capable of any mischief."

Can THE HOSPITAL, the sphinx of knowledge, kindly inform me, as an old and most interested reader of it, who the lady referred to can be?—Yours faithfully,

"IN TERROR."

